# The Outcomes of the Initial Misclassification of Undifferentiated Hypotension in the Emergency Department: A Prospective Observational Study

**DOI:** 10.3390/jcm13175293

**Published:** 2024-09-06

**Authors:** Jr-Jiun Lin, Wei-Ting Chen, Hooi-Nee Ong, Chi-Sheng Hung, Wei-Tien Chang, Chien-Hua Huang, Min-Shan Tsai

**Affiliations:** 1 Department of Emergency Medicine, National Taiwan University Medical College and Hospital, Taipei 100225, Taiwan; 2 Department of Internal Medicine, National Taiwan University Medical College and Hospital, Taipei 100225, Taiwan

**Keywords:** classification, emergency department, outcome, shock, undifferentiated hypotension

## Abstract

**Background:** Managing shock, a life-threatening emergency, is challenging. The influence of the initial misclassification of undifferentiated hypotension (UH) in the emergency department (ED) on patients’ outcomes remains uninvestigated. The aim of this study was to investigate whether the initial misclassification of UH in the ED affects patients’ clinical outcomes. **Materials and Methods:** This prospective observational study enrolled 270 non-traumatic adult patients with UH who had visited the ED of National Taiwan University Hospital between July 2020 and January 2022. The patients were divided into same-diagnosis and different-diagnosis groups, depending on the consistency between the initial and final classifications of shock. The outcome was survival to discharge. The clinical variables, management, and outcomes were compared between the groups. **Results:** A total of 39 of 270 patients (14.4%) were in the different-diagnosis group. Most misclassified patients were initially diagnosed as having hypovolemic shock (HS, *n* = 29) but finally diagnosed as having distributive shock (DS, *n* = 28) or cardiogenic shock (*n* = 1). When compared with the same-diagnosis group, the different-diagnosis group had higher hospitalization (94.9% vs. 81.4%, *p* = 0.023) but lower ED discharge (5.1% vs. 16.5%, *p* = 0.046) rates. Logistic regression analysis showed that the HS initially diagnosed was associated with an increased risk of misclassification (odds ratio [OR] = 14.731, 95% confidence interval [CI] = 3.572–60.749, *p* < 0.001). However, the survival to discharge did not differ between the two groups. DS, when finally diagnosed instead of the initial misclassification, was associated with in-hospital mortality (OR = 0.317, 95%CI = 0.124–0.810, *p* = 0.016). **Conclusions:** The misclassification of UH in the ED is not rare, particularly in patients with DS, who are likely to be initially misdiagnosed with HS. Although misclassification may increase hospitalization and decrease ED discharge, it does not affect survival to discharge.

## 1. Introduction

Shock, a life-threatening medical emergency caused by the inequality of oxygen supply and demand, is reversible initially but rapidly becomes irreversible, causing multiorgan failure and death [1,2,3,4,5]. Etiologically, shock is classified into cardiogenic shock (CS), hypovolemic shock (HS), obstructive shock (OS), and distributive shock (DS) [1,2,3,4,5]. The assessment and management of patients with shock remain challenging for emergency and critical care specialists, who need to appropriately treat diverse and variable clinical manifestations of shock through immediate evaluation, accurate diagnosis, precise etiological classification, and continuous maintenance of patients’ medical conditions, thereby preventing the development of reversible shock to irreversible organ failure or even death.

In a university hospital’s emergency department (ED) in Denmark, the average annual incidence of shock in adults was 63.2 cases per 100,000 population, increasing by 2.6% annually from 2000 to 2011, with a 7-day mortality rate of 23.1% and a 90-day mortality rate of approximately 40.7% [6]. The most common etiologies of shock in the ED in Denmark was DS (50.6%), followed by HS (30.8%), CS (14.0%), other conditions (3.7%), and OS (0.9%) [7]. A European multicenter study involving adults with shock who required vasopressors demonstrated that the most frequent type of shock was septic shock (62.2%), followed by CS (16.7%) and HS (15.7%); the least common type was OS (2%) [1]. In the ED, patients with shock or hypotension may fail to offer a detailed history initially and require resuscitation, making it challenging for the first-line physicians to adequately evaluate and classify the etiology of shock and ensure the proper management. The use of point-of-care ultrasound (PoCUS) on patients with shock has improved the clinical diagnostic accuracy for shock to 80–89% [8,9,10,11]. However, some studies have demonstrated that PoCUS does not improve survival and may worsen prognosis in hypotensive or critically ill patients in the ED [12,13].

Further examination and continuous patient management facilitate the accurate classification of shock type by the time of discharge. Studies have demonstrated the serious consequences of initial misdiagnosis; nonetheless, diagnostic errors are not uncommon in the ED [14,15,16,17]. However, the prevalence of the misclassification of shock and its effects on patients’ clinical outcomes remain unclear. Therefore, the current study aimed to investigate whether the initial misclassification of undifferentiated hypotension in the ED affects patients’ clinical outcomes.

## 2. Materials and Methods

### 2.1. Study Design and Patient Enrollment

The primary objective of this study was to explore whether the initial misclassification of undifferentiated hypotension in the ED has a significant impact on the clinical outcomes of patients. This prospective observational study was conducted in the ED of National Taiwan University Hospital (NTUH), a 2500-bed medical center that provides both primary and tertiary care in Taipei [18]. NTUH provides 24 h emergency care, and its ED has an average number of 300 visits per day. Critical patients are initially treated and constantly monitored in the critical area of the ED by experienced senior residents and attending physicians. The primary care physicians in the critical area of the ED may reserve the decisions to follow the standard operating protocols of the ED and might adjust the examinations and management according to the specific needs of each patient. This study was approved by the Institutional Review Board (IRB) of NTUH (IRB number: 202005121RINB; ClinicalTrials.gov Identifier: NCT04478045), and the written informed consents from all subjects and/or their legally authorized representative were obtained.

**Figure 1:**
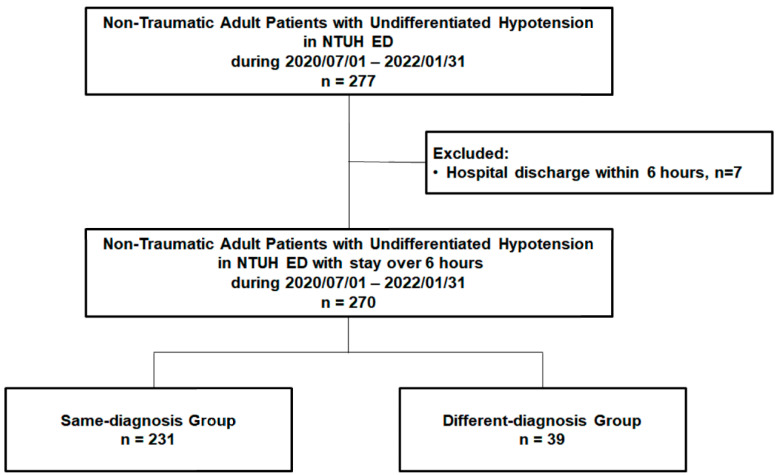
The flow chart of patient enrollment. ED: emergency department; NTUH: National Taiwan University Hospital.

Patients with undifferentiated hypotension were initially managed and observed by experienced senior residents and attending physicians in the critical area of the ED, who were blinded to the current study. They were then recruited, informed, and enrolled in the study by the staff of this study group and provided their consent. Usually, 4 to 5 patients with undifferentiated hypotension were admitted to the critical area of the ED each day, and 2 to 3 of these patients were recruited. Approximately 15–20% of the recruited patients were informed of the study and enrolled during the study period with their informed consent. The undifferentiated hypotension in the current study was defined as systolic blood pressure of <90 mmHg with a superimposed lactic acid level of >2.2 mmol/L or a clinical presentation of inadequate/insufficient tissue perfusion, as evaluated by ED physicians. Patients who experienced trauma, were pregnant, were aged <18 years, or had a do-not-resuscitate status were excluded from this study. Between July 2020 and January 2022, 277 non-traumatic adult patients visited the ED and were diagnosed as having undifferentiated hypotension. After excluding patients discharged within 6 h (*n* = 7), we included 270 non-traumatic adult patients with undifferentiated hypotension (Figure 1).

### 2.2. Data Collection

The following data were prospectively collected from the patients’ medical records: baseline characteristics, pre-existing comorbidities, clinical manifestations, physical examinations and laboratory results, initial diagnosis by ED physicians, clinical events and management during ED stay and hospitalization, and final diagnosis. Malignancy was defined as active or stable cancer. The Charlson comorbidity index (CCI) [19] and Acute Physiology and Chronic Health Evaluation II (APACHE II) scores [20] were used to evaluate comorbidity and clinical severity. The patients’ vital signs, clinical features, examinations, and laboratory results when presenting hypotension were evaluated by primary care physicians who were blinded to the current study. For assessing the cause and classification of hypotension, PoCUS was performed by trained and qualified doctors at the critical area, who were also blinded to this study. Fluid challenge was defined as the initial administration of >250 mL of intravenous crystalloid solutions in this study. A response to fluid challenge was defined as an increase >10 mmHg in systolic blood pressure after fluid challenge. The vasopressors and inotropes included dopamine, norepinephrine, epinephrine, vasopressin, or dobutamine. Patients with high respiratory support were defined when patients required bilevel positive airway pressure, high-flow oxygen therapy, or endotracheal intubation [21]. The definition of transfusion was the supplementation of blood products, including packed red blood cells, platelets, fresh frozen plasma, or cryoprecipitates. Emergent renal replacement therapy (RRT), such as sustained low-efficiency dialysis, continuous veno-venous hemofiltration, or hemodialysis, was indicated for refractory fluid overload with respiratory distress, severe electrolyte imbalance or metabolic acidosis, or overt uremic symptoms [21].

### 2.3. Outcome Measurements and Patient Grouping

The primary outcome was survival to hospital discharge. The main etiology of undifferentiated hypotension was evaluated and classified by ED physicians and by the primary care physicians in the intensive care unit (ICU) or ward separately, both who were blinded to the present study. The enrolled patients were divided into two groups—same-diagnosis and different-diagnosis groups—based on whether their shock type was misclassified (according to the consistency in shock classification between ED and ICU/ward physicians).

### 2.4. Statistical Analysis

We assumed that 80% of shock patients at the ED with the same diagnosis had an in-hospital mortality rate of 15%, whereas the 20% ones with a different diagnosis had a mortality rate of 35%. When the alpha level was 0.05 and the power was 0.85, the estimated sample size was about 270. Continuous variables with an approximately normal distribution between the two groups were presented as mean ± standard deviation values and compared using an independent *t* test. Continuous variables without a normal distribution between the two groups were presented as median (interquartile range [IQR]) and compared using the Mann–Whitney U test. Categorical variables were presented as number and percentage values and compared using the chi-squared test. A multiple logistic regression analysis, adjusted for the statistically significant variables of between-group differences in univariate analysis, was performed to identify the correlations between the independent variables and outcomes. Survival curves were plotted through the Kaplan–Meier analysis and compared between groups by using the log-rank test [22]. Statistical significance was set at *p* < 0.05. All statistical analyses were performed using Statistical Package for Social Sciences Statistics for Windows, version 21.0 (IBM Corp., Armonk, NY, USA).

## 3. Results

Of the enrolled 270 patients (mean age: 68.77 ± 15.34 years; men: 166 [61.5%]), 231 (85.6%) were included in the same-diagnosis group and 39 (14.4%) in the different-diagnosis group (Figure 1).

**Figure 2:**
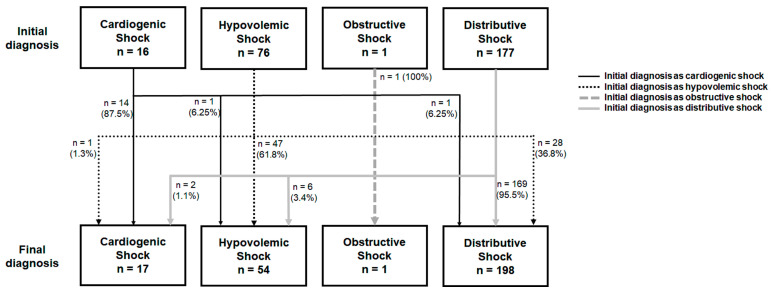
The numbers of patients with different types of shock between initial diagnosis and final diagnosis.

The baseline characteristics, clinical manifestations, and the PoCUS findings between the groups are listed in Table 1, and the management, outcomes, and classifications of shock between the groups were listed in Table 2. The different-diagnosis group had higher proportions of patients with a medical history of hypertension (53.8% vs. 37.2%, *p* = 0.050) and lower pulse rates when initially presenting with undifferentiated hypotension (99.86 ± 23.67/min vs. 108.95 ± 25.56/min, *p* = 0.044) than the same-diagnosis group. The CCI, the highest APACHE II score during hospitalization, and PoCUS findings did not differ between the two groups (Table 1). The management methods, including fluid challenge, the use of vasopressors and inotropes, and respiratory support were similar in these two groups (Table 2). The different-diagnosis group had higher proportions of patients initially diagnosed as having HS (74.4% vs. 20.3%, *p* < 0.001) and lower proportions of patients initially diagnosed as having DS (20.5% vs. 73.2%, *p* < 0.001) than the same-diagnosis group (Table 2). Most patients with misclassification were initially diagnosed as having hypovolemic shock (*n* = 29, 74.4%), but finally diagnosed as having distributive shock (*n* = 28) or cardiogenic shock (*n* = 1) (Figure 2). The multiple logistic regression analysis revealed that an initial diagnosis of HS (odds ratio [OR] = 14.731; 95% confidence interval [CI] = 3.572–60.749; *p* < 0.001) was significantly associated with an altered classification of shock (Table 3).

For the patients initially diagnosed with HS (Supplementary Table S1), those misclassified as having HS (*n* = 29) showed a higher prevalence of diabetes mellitus, bedridden status, altered conscious state, cachexia, dry skin turgor, inotrope use, and ICU admission than those with a consistent HS diagnosis (*n* = 47); additionally, these misclassified patients also had higher body temperatures and APACHE II scores during hospitalization than those consistently diagnosed with HS. Notably, many patients who misclassified HS exhibited inferior vena cava (IVC) collapse level of >50% when presenting hypotension, but only approximately one-third of them ultimately responded to fluid challenge. In our study, the sensitivity, specificity, and accuracy were, respectively, 82.4%, 99.2%, and 98.2% for CS; 87.0%, 86.6%, and 86.7% for HS; 100.0%, 100.0%, and 100.0% for OS; and 85.4%, 88.9%, and 86.3% for DS.

When compared with the same-diagnosis group, the different-diagnosis group had a higher percentage of hospitalization (94.9% vs. 81.4%, *p* = 0.023) but a lower percentage of ED discharge (5.1% vs. 16.5%, *p* = 0.046). These two groups did not differ in ICU admission, survival to discharge, and the hospital length of stay (Table 2). The survival curves did not differ between the two groups (Supplementary Figure S1). The 7-day and 30-day survival rates in the same-diagnosis group were 93.5% and 84.8%, respectively, whereas the 7-day and 30-day survival rates in the different-diagnosis group were 92.3% and 82.1%, respectively. A total of 207 (76.7%) patients survived to hospital discharge, and 63 (23.3%) patients failed. Supplementary Tables S2 and S3 indicate the clinical variables grouped by survival to discharge or not. No significant difference in the misclassification of hypotension was noted between the survivor and non-survivor groups. The multiple logistic regression analysis revealed that DS as the final diagnosis (OR = 0.317; 95% CI = 0.124–0.810; *p* = 0.016) was significantly associated with the lower possibility of survival to discharge (Table 4).

## 4. Discussion

This prospective observational study enrolled 270 non-traumatic adult patients who presented with undifferentiated hypotension in our ED and identified that the initial misclassification of shock was not rare. Most patients with misclassification were initially diagnosed as having hypovolemic shock, but the initial misclassification did not change the patients’ survival to discharge.

Diagnostic errors are not uncommon in the ED and may cause further complications or mortalities [16,17]. Given the heterogeneous symptoms and pathophysiology of shock, the etiologies of shock in some patients could be misclassified in the ED. In our study, 85.6% of all shock cases in the ED were accurately classified, similar to the accuracy of 89% observed in another study which included non-traumatic ED patients with undifferentiated hypotension [15]. In our study, HS and DS (particularly septic shock) were the most common types of shock, consistent with the finding of other studies [1,7], and DS was often initially misclassified as HS. In addition to history-taking and physical examination of patients with undifferentiated hypotension in the ED, PoCUS is currently extensively applied in clinical settings for rapid evaluations and quick diagnoses in the ED; this helps ED physicians to further stabilize and appropriately treat patients initially [8,9,10,11]. However, Atkinson PR et al. reported that PoCUS did not significantly improve the survival of patients with undifferentiated hypotension in the ED [12], and Mosier JM et al. indicated that the PoCUS results obtained in the ED might be associated with increased mortality in critically ill patients [13]. Our results showed that an initial misclassification of shock with PoCUS did not significantly affect survival to discharge in patients with undifferentiated hypotension in the ED.

A systematic review and meta-analysis conducted by Yoshida T et al. revealed different sensitivities and specificities of PoCUS among patients with shock (78% and 96% for CS, 90% and 92% for HS, 82% and 98% for OS, and 79% and 96% for DS) [23]. In our study, both sensitivity and specificity for OS were 100.0%, and Yoshida T et al. also indicated that PoCUS usually exhibited a high diagnostic accuracy for OS [23]. However, the specificity was only 89% for DS in our study, and DS was a major type prone to misclassification in the ED. When cardiac output or contractility reduced, the structural obstruction of the heart or large vessels, or distended IVC were observed in patients with shock, and CS or OS could be correctly and rapidly diagnosed [23,24,25]. For the patients with shock who were initially misclassified as having HS or DS, PoCUS might indicate a flat IVC with or without a hypercontractile heart [24,25]. In our study, the 28 patients who were initially misclassified as having HS and were later diagnosed as having DS exhibited no typical objective symptoms or signs of septic shock, such as fever, tachycardia, or leukocytosis [4,5,7]; furthermore, among these misclassified patients, increased proportions of patients were with altered mental status and cachexia, required inotrope use, and underwent ICU admission, indicating a severe shock status necessitating urgent treatment. Many of them reasonably indicated an IVC collapse level of >50% via PoCUS. This likely explained why they were initially misclassified as having HS. Only 10 patients from this patient population exhibited hemodynamic responses after fluid challenges. One of the possible hints for the discrimination of HS and DS is that fluid responsiveness is better for patients with HS; however, this requires time to observe and monitor, which may be limited in the ED.

Although taking a detailed history and monitoring patients’ responsiveness to fluid challenge and medications might ensure an accurate diagnosis, ED physicians might find it challenging to accomplish this in the crowded and busy ED. The number of ED visits has been gradually increasing worldwide [26,27]. Kim DU et al. demonstrated that ED overcrowding increased the rate of return visits within 72 h of ED discharge, likely because of medical mistakes, previous misdiagnosis, postponed diagnosis, and delay in alerting about disease progression [28]. ED overcrowding affects ED physicians’ accuracy in classifying the etiology of shock and the timely recognition of patient response to management strategies. In our study, approximately 92.6% of all patients underwent fluid challenge and 94.4% were prescribed antibiotics. For the patients who were initially misclassified as having HS and confirmed as having DS afterwards, the high prevalence of fluid challenge and empirical antibiotics in the ED might cover the misclassification. For the patients who were initially misclassified as having DS and diagnosed as having HS later, the initial fluid challenge under close hemodynamic monitor might not have led to severe outcomes. These findings suggest that ED physicians treating undifferentiated hypotensive patients may face the possibility of the misclassification of hypotension due to scant medical history, limited resources, and time constraints. Prompt and comprehensive critical management according to patients’ personalized dynamic clinical status may remedy the misclassification of hypotension in the ED.

This study has several limitations. First, the observational design precluded the verification of the clinical variables not listed in this study with outcomes. Second, the results of PoCUS, which are highly dependent on the accuracy and ability of the performers, might have caused information bias. Third, because our study outcome was based on survival to discharge rather than survival to a fixed time point after presenting shock, a categorical bias might have occurred because hospitalization duration might be influenced not only by shock but also by other factors. Finally, because of the diversities in patient characteristics, clinical manifestation, and management across health insurance systems, hospitals, and countries, further larger-scale prospective studies are needed to assess the generalizability of our findings.

## 5. Conclusions

The misclassification of undifferentiated hypotension in the ED is not rare, particularly in patients with DS, who are likely to receive an initial misdiagnosis of HS. Diagnostic errors might be due to atypical objective symptoms or signs, similar flat IVC results from PoCUS, or limited time to observe fluid responsiveness in the ED. Although misclassification may increase hospitalization and decrease ED discharge, it does not affect survival to hospital discharge.

## Figures and Tables

**Table 1:** Baseline characteristics, initial clinical features, and PoCUS findings between the groups.

	Total Patients *n* (%)		Same-Diagnosis *n* (%)		Different-Diagnosis *n* (%)		*p*-Value
	*n* = 270		*n* = 231	(85.6%)	*n* = 39	(14.4%)	
***Baseline characteristics***							
Age (year)	68.77 ± 15.34		68.74 ± 15.15		68.95 ± 16.67		0.939
>65	157	(58.1%)	136	(58.9%)	21	(53.8%)	0.556
Sex (male)	166	(61.5%)	146	(63.2%)	20	(51.3%)	0.157
Diabetes mellitus	80	(29.6%)	64	(27.7%)	16	(41.0%)	0.092
Hypertension	107	(39.6%)	86	(37.2%)	21	(53.8%)	0.050
CAD	35	(13.0%)	31	(13.4%)	4	(10.3%)	0.404
Heart failure	18	(6.7%)	16	(6.9%)	2	(5.1%)	0.502
COPD	13	(4.8%)	11	(4.8%)	2	(5.1%)	0.585
ESRD	24	(8.9%)	22	(9.5%)	2	(5.1%)	0.293
Liver cirrhosis	16	(5.9%)	12	(5.2%)	4	(10.3%)	0.186
Peptic ulcer disease history	12	(4.4%)	10	(4.3%)	2	(5.1%)	0.539
CVA	14	(5.2%)	13	(5.6%)	1	(2.6%)	0.372
Bed ridden	14	(5.2%)	11	(4.8%)	3	(7.7%)	0.328
Malignancy	130	(48.1%)	115	(49.8%)	15	(38.5%)	0.191
Charlson comorbidity index	4.67 ± 2.36		4.67 ± 2.34		4.69 ± 2.51		0.950
***Vital signs and laboratory results***							
GCS 13–15	215	(79.6%)	186	(80.5%)	29	(74.4%)	0.377
Body temperature (°C)	37.34 ± 1.28		37.36 ± 1.33		37.20 ± 0.95		0.465
Pulse rate (/min)	107.69 ± 25.46		108.95 ± 25.56		99.86 ± 23.67		0.044
Respiratory rate (/min)	20.95 ± 3.54		21.05 ± 3.64		20.37 ± 2.85		0.274
SBP (mmHg)	87.21 ± 23.27		87.14 ± 23.70		87.71 ± 20.53		0.891
DBP (mmHg)	54.56 ± 13.64		54.58 ± 13.52		54.43 ± 14.58		0.950
Highest APACHE II	22.60 ± 7.16		22.36 ± 7.17		24.00 ± 7.12		0.385
pH	7.37 ± 0.46		7.34 ± 0.49		7.37 ± 0.11		0.679
HCO_3_^−^ (mmol/L)	21.72 ± 8.02		21.55 ± 5.92		22.74 ± 15.55		0.392
Lactic acid (mmol/L)	3.93 ± 2.62		3.90 ± 2.55		4.09 ± 3.03		0.672
WBC (K/μL)	11.17 ± 7.78		11.05 ± 7.79		11.89 ± 7.81		0.535
Hb (g/dL)	10.89 ± 2.89		10.88 ± 2.88		11.00 ± 3.03		0.807
Total bilirubin (mg/dL), median (IQR)	1.55 (0.59–1.45)		1.59 (0.59–1.44)		1.29 (0.63–1.50)		0.517
Creatinine (mg/dL), median(IQR)	2.30 (1.00–2.60)		2.28 (1.00–2.55)		2.42 (1.00–2.83)		0.279
Troponin-T (ng/L), median(IQR)	158.67 (24.57–126.53)		145.74 (25.59–124.55)		228.70 (20.66–150.10)		0.777
NT-proBNP (pg/mL), median(IQR)	4358.82 (565.23–4429.25)		4531.62 (688.80–4496.00)		3552.43 (193.35–2006.25)		0.036
***PoCUS***							
Heart							
LVEF (%)	38.84 ± 19.79		38.71 ± 20.68		40.00 ± 14.14		0.933
Abnormal wall motion	8	(3.0%)	6	(2.6%)	2	(5.1%)	0.325
Pericardial effusion	28	(10.4%)	27	(11.7%)	1	(2.6%)	0.062
IVC collapse > 50%	120	(44.4%)	102	(44.2%)	18	(46.2%)	0.816
Pleural effusion	48	(17.8%)	44	(19.0%)	4	(10.3%)	0.133
Moderate or large amount	16	(5.9%)	14	(6.1%)	2	(5.1%)	0.585
Ascites	45	(16.7%)	39	(16.9%)	6	(15.4%)	0.816
Moderate or large amount	17	(6.3%)	15	(6.5%)	2	(5.1%)	0.543

APACHE: acute physiology and chronic health evaluation; CAD: coronary artery disease; COPD: chronic obstructive pulmonary disease; CVA: cerebrovascular accident; DBP: diastolic blood pressure; ESRD: end-stage renal disease; GCS: Glasgow Coma Scale; Hb: hemoglobin; HCO_3_^−^: bicarbonate; IQR, interquartile range; IVC: inferior vena cava; LVEF: left ventricular ejection fraction; NT-proBNP: n-terminal pro-brain natriuretic peptide; PoCUS: point-of-care ultrasound; SBP: systolic blood pressure; WBC: white blood cell.

**Table 2:** Management, outcomes, and diagnosis between the groups.

	Total Patients *n* (%)		Same-Diagnosis *n* (%)		Different-Diagnosis *n* (%)		*p*-Value
	*n* = 270		*n* = 231	(85.6%)	*n* = 39	(14.4%)	
***Management***							
Fluid challenge	250	(92.6%)	214	(92.6%)	36	(92.3%)	0.575
Response to fluid challenge	101	(37.4%)	87	(37.7%)	14	(35.9%)	0.833
Inotropes	195	(72.2%)	167	(72.3%)	28	(71.8%)	0.949
Multiple (≧2)	50	(18.5%)	44	(19.0%)	6	(15.4%)	0.586
Respiratory support							
Room air or low	192	(71.1%)	166	(71.9%)	26	(66.7%)	0.508
High	78	(28.9%)	65	(28.1%)	13	(33.3%)	
Antibiotics	255	(94.4%)	218	(94.4%)	37	(94.9%)	0.628
Transfusion	92	(34.1%)	80	(34.6%)	12	(30.8%)	0.638
Emergent RRT	69	(25.6%)	60	(26.0%)	9	(23.1%)	0.701
***Outcomes***							
ED Disposition							
Hospitalization	225	(83.3%)	188	(81.4%)	37	(94.9%)	0.023
ICU admission	124	(45.9%)	106	(45.9%)	18	(46.2%)	0.975
Duration of hospitalization (day)	23.57 ± 23.62		24.26 ± 24.62		20.05 ± 17.46		0.322
Discharge from ED	40	(14.8%)	38	(16.5%)	2	(5.1%)	0.046
Duration of ED stay (day)	2.48 ± 1.53		2.50 ± 1.56		2.01 ± 0.31		0.664
Death in the ED	5	(1.9%)	5	(2.2%)	0	(0%)	0.456
Duration of ED stay (day)	2.15 ± 2.40		2.15 ± 2.40				
Survival to discharge	207	(76.7%)	178	(77.1%)	29	(74.4%)	0.713
Length of stay (day)	20.05 ± 22.96		20.21 ± 23.79		19.12 ± 17.47		0.786
***Initial Diagnosis at the ED***							
Cardiogenic shock	16	(5.9%)	14	(6.1%)	2	(5.1%)	0.585
Hypovolemic shock	76	(28.1%)	47	(20.3%)	29	(74.4%)	<0.001
Obstructive shock	1	(0.4%)	1	(0.4%)	0	(0.0%)	0.856
Distributive shock	177	(65.6%)	169	(73.2%)	8	(20.5%)	<0.001
***Final Diagnosis***							
Cardiogenic shock	17	(6.3%)	14	(6.1%)	3	(7.7%)	0.457
Hypovolemic shock	54	(20.0%)	47	(20.3%)	7	(17.9%)	0.729
Obstructive shock	1	(0.4%)	1	(0.4%)	0	(0.0%)	0.856
Distributive shock	198	(73.3%)	169	(73.2%)	29	(74.4%)	0.876

ED: emergency department; ICU: intensive care unit; RRT: renal replacement therapy.

**Table 3:** The association between the clinical factors and the misclassification of shock.

	Odds Ratio	95% Confidence Interval	*p*-Value
Hypertension	2.266	0.651–7.886	0.198
Pulse rate	0.983	0.959–1.008	0.173
NT-proBNP	1.000	1.000–1.000	0.388
Initial diagnosis at the ED			
Distributive shock	Reference	Reference	-
Hypovolemic shock	14.731	3.572–60.749	<0.001
Other shock	0.699	0.064–7.596	0.769

ED: emergency department; NT-proBNP: n-terminal pro-brain natriuretic peptide.

**Table 4:** The association between the clinical factors and survival to discharge.

	Odds Ratio	95% Confidence Interval	*p*-Value
Charlson comorbidity index ≧ 4	0.374	0.162–0.860	0.021
Glasgow Coma Scale 13–15	1.435	0.657–3.134	0.365
Lactic acid ≧ 4 mmol/L	0.687	0.350–1.349	0.276
Moderate or large amount of ascites	0.323	0.100–1.046	0.059
Multiple inotropes	0.243	0.107–0.551	0.001
High respiratory support	0.281	0.133–0.594	0.001
Distributive shock as the final diagnosis	0.317	0.124–0.810	0.016

## Data Availability

The datasets used and/or analyzed during the current study are available from the corresponding author upon reasonable request.

## Supplementary Materials

The following supporting information can be downloaded at: https://www.mdpi.com/article/10.3390/jcm13175293/s1, Table S1: Baseline characteristics, initial clinical features, PoCUS findings, management, outcomes, and final diagnosis between the groups among the patients with initial diagnosis as HS; Table S2: Baseline characteristics, initial clinical features, and PoCUS findings between the survivors and non-survivors; Table S3: Management, outcomes, and diagnosis between the survivors and non-survivors; Figure S1: The survival curves of the same-diagnosis and different-diagnosis groups.

## Abbreviations

APACHE II	Acute Physiology and Chronic Health Evaluation II
CCI	Charlson comorbidity index
CI	Confidence interval
CS	Cardiogenic shock
DS	Distributive shock
ED	Emergency department
HS	Hypovolemic shock
ICU	Intensive care unit
IQR	Interquartile range
IRB	Institutional Review Board
IVC	Inferior vena cava
NTUH	National Taiwan University Hospital
OR	Odds ratio
OS	Obstructive shock
PoCUS	Point-of-care ultrasound
RRT	Renal replacement therapy
