# Supplementary material for: The Outcomes of the Initial Misclassification of Undifferentiated Hypotension in the Emergency Department: A Prospective Observational Study
Source: J Clin Med. 2024 Sep 6;13(17):5293. doi: 10.3390/jcm13175293 (PMC11396653; doi:10.3390/jcm13175293)
Supplement: Supplementary file 1 [file jcm-13-05293-s001.zip › 0827 Supplementary Figure S1.pdf]

Supplementary Figure S1. The survival curves of the same-diagnosis and different-diagnosis groups.

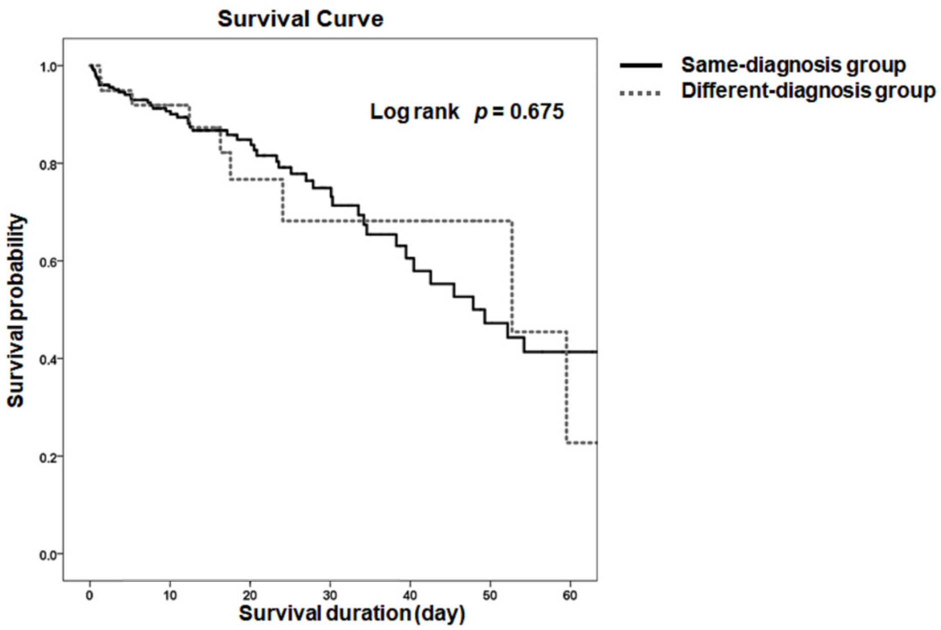

Number at risk

	0	10	20	30	40	50	60
Same-diagnosis group	231	148	79	43	23	17	11
Different-diagnosis group	39	25	12	8	6	3	1

Survival duration (day)
