# Supplementary material for: The Outcomes of the Initial Misclassification of Undifferentiated Hypotension in the Emergency Department: A Prospective Observational Study
Source: J Clin Med. 2024 Sep 6;13(17):5293. doi: 10.3390/jcm13175293 (PMC11396653; doi:10.3390/jcm13175293)
Supplement: Supplementary file 1 [file jcm-13-05293-s001.zip › 0827 supplementary table S1.pdf]

Supplementary Table S1. Baseline characteristics, initial clinical features, PoCUS findings, management, outcomes, and final diagnosis between the groups among the patients with initial diagnosis as HS.

	Total patients n (%) n=76	Same-diagnosis n (%) n=47 (61.8%)	Different-diagnosis n (%) n=29 (38.2%)	*p*-value
Baseline characteristics				
Age (year)	68.05 ± 15.87	67.07 ± 15.97	69.62 ± 15.85	0.500
> 65	40 (52.6%)	24 (51.1%)	16 (55.2%)	0.727
Sex (male)	43 (56.6%)	26 (55.3%)	17 (58.6%)	0.778
Diabetes mellitus	20 (26.3%)	8 (17.0%)	12 (41.4%)	0.019
Hypertension	33 (43.4%)	20 (42.6%)	13 (44.8%)	0.846
CAD	7 (9.2%)	6 (12.8%)	1 (3.4%)	0.171
Heart failure	5 (6.6%)	4 (8.5%)	1 (3.4%)	0.363
COPD	3 (3.9%)	2 (4.3%)	1 (3.4%)	0.677
ESRD	6 (7.9%)	4 (8.5%)	2 (6.9%)	0.584
Liver cirrhosis	8 (10.5%)	5 (10.6%)	3 (10.3%)	0.642
Peptic ulcer disease history	5 (6.6%)	3 (6.4%)	2 (6.9%)	0.637
CVA	2 (2.6%)	2 (4.3%)	0 (0.0%)	0.379
Bed ridden	3 (3.9%)	0 (0.0%)	3 (10.3%)	0.052
Malignancy	34 (44.7%)	23 (48.9%)	11 (37.9%)	0.349
Charlson Comorbidity Index	4.59 ± 2.42	4.40 ± 2.36	4.90 ± 2.53	0.392
Vital signs, Clinical features, and Laboratory results				
GCS 13 – 15	68 (89.5%)	47 (100.0%)	21 (72.4%)	<0.001
Body temperature (℃)	36.88 ± 0.88	36.70 ± 0.82	37.17 ± 0.89	0.021
Pulse rate (/min)	101.44 ± 22.19	102.77 ± 22.35	99.21 ± 22.15	0.506
Respiratory rate (/min)	20.14 ± 2.73	19.94 ± 2.38	20.48 ± 3.23	0.399
SBP (mmHg)	81.40 ± 16.90	77.13 ± 12.53	89.12 ± 20.92	0.003
Cachexia	12 (15.8%)	5 (10.6%)	7 (24.1%)	0.117
Dry skin turgor	43 (56.6%)	22 (46.8%)	21 (72.4%)	0.029
Highest APACHE II	23.41 ± 7.47	20.50 ± 7.79	25.83 ± 6.55	0.096
pH	7.36 ± 0.12	7.36 ± 0.11	7.37 ± 0.12	0.959
HCO_3^- (mmol/L)	22.26 ± 11.88	21.26 ± 5.80	23.87 ± 17.84	0.356
Lactic Acid (mmol/L)	3.73 ± 2.66	3.61 ± 2.23	3.92 ± 3.25	0.623
WBC (K/μL)	12.04 ± 6.90	12.45 ± 6.83	11.36 ± 7.07	0.508
Hb (g/dL)	10.42 ± 3.37	10.15 ± 3.53	10.86 ± 3.11	0.376
Total Bilirubin (mg/dL), median(IQR)	1.38(0.56-1.49)	1.41(0.52-1.47)	1.32(0.74-1.62)	0.199
Creatinine (mg/dL), median(IQR)	2.34(1.00-3.00)	2.14(0.95-2.75)	2.65(1.00-3.38)	0.254

Troponin-T (ng/L), median(IQR)	156.08(22.70-101.65)	61.44(23.36-77.29)	288.57(20.45-155.50)	0.427
NT-proBNP (pg/mL), median(IQR)	1823.95(245.10-1664.00)	1095.98(367.98-1357.25)	2607.93(207.85-2027.50)	0.793
PoCUS				
Heart				
LVEF (%)	50.50 ± 6.44	50.00 ± 8.16	51.50 ±2.12	0.821
Abnormal wall motion	1 (1.3%)	0 (0.0%)	1 (3.4%)	0.382
Pericardial effusion	5 (6.6%)	4 (8.5%)	1 (3.4%)	0.363
IVC collapse > 50%	40 (52.6%)	24 (51.1%)	16 (55.2%)	0.727
Pleural effusion	6 (7.9%)	4 (8.5%)	2 (6.9%)	0.584
Ascites	13 (17.1%)	9 (19.1%)	4 (13.8%)	0.393
Management				
Fluid challenge	72 (94.7%)	45 (95.7%)	27 (93.1%)	0.494
Response to fluid challenge	36 (47.4%)	26 (55.3%)	10 (34.5%)	0.077
Inotropes	35 (46.1%)	17 (36.2%)	18 (62.1%)	0.028
Multiple (≧2)	7 (9.2%)	5 (10.6%)	2 (6.9%)	0.456
Respiratory support				
Room air or low	59 (77.6%)	39 (83.0%)	20 (69.0%)	0.154
High	17 (22.4%)	8 (17.0%)	9 (31.0%)	
Antibiotics	69 (90.8%)	40 (85.1%)	29 (100.0%)	0.029
Transfusion	40 (52.6%)	20 (42.6%)	20 (69.0%)	0.025
Emergent RRT	19 (25.0%)	11 (23.4%)	8 (27.6%)	0.683
Outcomes				
ED disposition				
Hospitalization	56 (73.7%)	28 (59.6%)	28 (96.6%)	<0.001
ICU admission	25 (32.9%)	12 (25.5%)	13 (44.8%)	0.082
Duration of hospitalization (day)	19.32 ± 18.50	21.21 ± 19.60	17.43 ± 17.48	0.449
Discharge from ED	20 (26.3%)	19 (40.4%)	1 (3.4%)	<0.001
Duration of ED stay (day)	2.55 ± 1.37	2.59 ± 1.39	1.79	0.585
Death in the ED	0 (0.0%)			
Duration of ED stay (day)	-			
Survival to discharge	61 (80.3%)	41 (87.2%)	20 (69.0%)	0.052
Length of stay (day)	14.91 ± 17.51	13.68 ± 17.65	16.89 ± 17.41	0.442
Final Diagnosis				
Cardiogenic shock	1 (1.3%)	0 (0.0%)	1 (3.4%)	0.382
Hypovolemic shock	47 (61.8%)	47 (100.0%)	0 (0.0%)	<0.001
Obstructive shock	0 (0.0%)	0 (0.0%)	0 (0.0%)	
Distributive shock	28 (36.8%)	0 (0.0%)	28 (96.6%)	<0.001

APACHE: acute physiology and chronic health evaluation; CAD: coronary artery disease; COPD: chronic obstructive pulmonary disease; CVA: cerebrovascular accident; ED: emergency department; ESRD: end-stage renal disease; GCS: Glasgow Coma Scale; Hb: hemoglobin; HCO_3^-: bicarbonate; HS: hypovolemic shock; ICU: intensive care unit; IQR, interquartile range; IVC: inferior vena cava; LVEF: left ventricular ejection fraction; NT-proBNP: n-terminal pro-brain natriuretic peptide; PoCUS: point-of-care ultrasound; RRT: renal replacement therapy; SBP: systolic blood pressure; WBC: white blood cell.
