# Supplementary material for: The Outcomes of the Initial Misclassification of Undifferentiated Hypotension in the Emergency Department: A Prospective Observational Study
Source: J Clin Med. 2024 Sep 6;13(17):5293. doi: 10.3390/jcm13175293 (PMC11396653; doi:10.3390/jcm13175293)
Supplement: Supplementary file 1 [file jcm-13-05293-s001.zip › 0827 supplementary table S2.pdf]

Supplementary Table S2. Baseline characteristics, initial clinical features, and PoCUS findings between the survivors and non-survivors.

	Total patients n (%) n=270	Survivor n (%) n=207 (76.7%)	Non-survivor n (%) n=63 (23.3%)	*p*-value
Baseline characteristics				
Age (year)	68.77 ± 15.34	68.18 ± 5.84	70.73 ± 13.51	0.249
> 65	157 (58.1%)	114 (55.1%)	43 (68.3%)	0.063
Sex (male)	166 (61.5%)	128 (61.8%)	38 (60.3%)	0.828
Diabetes mellitus	80 (29.6%)	62 (30.0%)	18 (28.6%)	0.834
Hypertension	107 (39.6%)	91 (44.0%)	16 (25.4%)	0.008
CAD	35 (13.0%)	28 (13.5%)	7 (11.1%)	0.617
Heart failure	18 (6.7%)	8 (3.9%)	10 (15.9%)	0.001
COPD	13 (4.8%)	9 (4.3%)	4 (6.3%)	0.359
ESRD	24 (8.9%)	21 (10.1%)	3 (4.8%)	0.143
Liver cirrhosis	16 (5.9%)	14 (6.8%)	2 (3.2%)	0.234
Peptic ulcer disease history	12 (4.4%)	9 (4.3%)	3 (4.8%)	0.560
CVA	14 (5.2%)	8 (3.9%)	6 (9.5%)	0.076
Bed ridden	14 (5.2%)	6 (2.9%)	8 (12.7%)	0.002
Malignancy	130 (48.1%)	92 (44.4%)	38 (60.3%)	0.027
Charlson Comorbidity Index	4.67 ± 2.36	4.48 ± 2.36	5.29 ± 2.26	0.018
Vital signs and Laboratory results				
GCS 13 – 15	215 (79.6%)	173 (83.6%)	42 (66.7%)	0.004
Body temperature (°C)	37.34 ± 1.28	37.33 ± 1.28	37.38 ± 1.30	0.767
Pulse rate (/min)	107.69 ± 25.46	106.02 ± 24.81	113.24 ± 26.96	0.050
Respiratory rate (/min)	20.95 ± 3.54	20.58 ± 2.98	22.16 ± 4.78	0.002
SBP (mmHg)	87.21 ± 23.27	86.68 ± 22.69	88.98 ± 25.23	0.499
Highest APACHE II	22.60 ± 7.16	20.93 ± 6.09	26.03 ± 8.00	<0.001
pH	7.37 ± 0.46	7.34 ± 0.52	7.37 ± 0.13	0.647
HCO_3^- (mmol/L)	21.72 ± 8.02	22.20 ± 8.40	20.14 ± 6.42	0.073
Lactic Acid (mmol/L)	3.93 ± 2.62	3.67 ± 2.34	4.76 ± 3.27	0.004
WBC (K/μL)	11.17 ± 7.78	11.37 ± 7.64	10.52 ± 8.27	0.453
Hb (g/dL)	10.89 ± 2.89	11.04 ± 2.87	10.40 ± 2.93	0.123
Total Bilirubin (mg/dL), median(IQR)	1.55(0.59-1.45)	1.44(0.60-1.47)	1.87(0.59-1.28)	0.190
Creatinine (mg/dL), median(IQR)	2.30(1.00-2.60)	2.32(1.00-2.65)	2.25(1.08-2.53)	0.768
Troponin-T (ng/L), median(IQR)	158.67(24.57-126.53)	167.56(23.83-122.15)	130.84(26.74-141.85)	0.217
NT-proBNP (pg/mL), median(IQR)	4358.82(565.23-4429.25)	3467.70(383.38-3466.25)	6497.50(1157.50-7836.75)	0.005
PoCUS				

Heart							
LVEF (%)	38.84 ± 19.79		35.00 ± 21.60		47.17 ± 12.97		0.222
Abnormal wall motion	8	(3.0%)	5	(2.4%)	3	(4.8%)	0.279
Pericardial effusion	28	(10.4%)	19	(9.2%)	9	(14.3%)	0.244
IVC collapse > 50%	120	(44.4%)	90	(43.5%)	30	(47.6%)	0.562
Pleural effusion	48	(17.8%)	35	(16.9%)	13	(20.6%)	0.498
Moderate or large amount	16	(5.9%)	11	(5.3%)	5	(7.9%)	0.440
Ascites	45	(16.7%)	25	(12.1%)	20	(31.7%)	<0.001
Moderate or large amount	17	(6.3%)	8	(3.9%)	9	(14.3%)	0.003

APACHE: acute physiology and chronic health evaluation; CAD: coronary artery disease; COPD: chronic obstructive pulmonary disease; CVA: cerebrovascular accident; ESRD: end-stage renal disease; GCS: Glasgow Coma Scale; Hb: hemoglobin; HCO_3^-: bicarbonate; IQR, interquartile range; IVC: inferior vena cava; LVEF: left ventricular ejection fraction; NT-proBNP: n-terminal pro-brain natriuretic peptide; PoCUS: point-of-care ultrasound; SBP: systolic blood pressure; WBC: white blood cell.
