# Supplementary material for: The Outcomes of the Initial Misclassification of Undifferentiated Hypotension in the Emergency Department: A Prospective Observational Study
Source: J Clin Med. 2024 Sep 6;13(17):5293. doi: 10.3390/jcm13175293 (PMC11396653; doi:10.3390/jcm13175293)
Supplement: Supplementary file 1 [file jcm-13-05293-s001.zip › 0827 supplementary table S3.pdf]

Supplementary Table S3. Management, outcomes, and diagnosis between the survivors and non-survivors.

	Total patients n (%) n=270		Survivor n (%) n=207 (76.7%)		Non-survivor n (%) n=63 (23.3%)		*p*-value
Management							
Fluid challenge	250	(92.6%)	188	(90.8%)	62	(98.4%)	0.030
Response to fluid challenge	101	(37.4%)	71	(34.3%)	30	(47.6%)	0.056
Inotropes	195	(72.2%)	140	(67.6%)	55	(87.3%)	0.002
Multiple (≧2)	50	(18.5%)	23	(11.1%)	27	(42.9%)	<0.001
Respiratory support							
Room air or low	192	(71.1%)	167	(80.7%)	25	(39.7%)	<0.001
High	78	(28.9%)	40	(19.3%)	38	(60.3%)	
Antibiotics	255	(94.4%)	192	(92.8%)	63	(100.0%)	0.016
Transfusion	92	(34.1%)	66	(31.9%)	26	(41.3%)	0.169
Emergent RRT	69	(25.6%)	45	(21.7%)	24	(38.1%)	0.009
Outcomes							
ED disposition							
Hospitalization	225	(83.3%)	167	(80.7%)	58	(92.1%)	0.034
ICU admission	124	(45.9%)	83	(40.1%)	41	(65.1%)	<0.001
Duration of hospitalization (day)	23.57 ± 23.62		22.36 ± 22.48		27.05 ± 26.51		0.193
Discharge from ED	40	(14.8%)	40	(19.3%)	0	(0.0%)	<0.001
Duration of ED stay (day)	2.48 ± 1.53		2.48 ± 1.53		-		
Death in the ED	5	(1.9%)	0	(0.0%)	5	(7.9%)	0.001
Duration of ED stay (day)	2.15 ± 2.40		-		2.15 ± 2.40		
Length of stay (day)	20.05 ± 22.96		18.52 ± 21.67		25.08 ± 26.32		0.047
Initial diagnosis at the ED							
Cardiogenic shock	16	(5.9%)	15	(7.2%)	1	(1.6%)	0.077
Hypovolemic shock	76	(28.1%)	61	(29.5%)	15	(23.8%)	0.382
Obstructive shock	1	(0.4%)	1	(0.5%)	0	(0.0%)	0.767
Distributive shock	177	(65.6%)	130	(62.8%)	47	(74.6%)	0.084
Final diagnosis							
Cardiogenic shock	17	(6.3%)	16	(7.7%)	1	(1.6%)	0.061
Hypovolemic shock	54	(20.0%)	47	(22.7%)	7	(11.1%)	0.044
Obstructive shock	1	(0.4%)	1	(0.5%)	0	(0.0%)	0.767
Distributive shock	198	(73.3%)	143	(69.1%)	55	(87.3%)	0.004
Misclassification of shock	39	(14.4%)	29	(14.0%)	10	(15.9%)	0.713

ED: emergency department ICU: intensive care unit; RRT: renal replacement therapy.
